# Are we there yet with XNA aptamers?

**DOI:** 10.1039/d5ra05395a

**Published:** 2026-04-29

**Authors:** Santiago Chaillou, Saphyra Thonon, Sten Reynders, Eveline Lescrinier, Vitor B. Pinheiro

**Affiliations:** a KU Leuven, Rega Institute for Medical Research, Department of Pharmaceutical and Pharmacological Sciences Herestraat, 49 Leuven 3000 Belgium v.pinheiro@kuleuven.be

## Abstract

Since their original development, aptamers (or nucleic acid oligomers of defined sequence that are capable of high-affinity and high-specificity binding to a target) have been heralded as possible and promising new therapeutic modalities. Increasingly versatile and customizable aptamer selection (SELEX) processes, alongside incorporation of novel synthetic nucleic acid (XNA) chemistries, have played a key role in the development of therapeutically relevant molecules. Nonetheless, despite the significant progress in the last 40 years, clinical applications of aptamers remain a nascent field. This review summarises some of the key developments in the field in the last 40 years, highlighting the progress made in aptamer selection, XNA chemistries and computational analyses. We discuss the intricacies and limitations of the current gold standard in aptamer SELEX and underscore the added complexities of XNA incorporation. Reflecting on the distinction between the antibody and aptamer fields, we advocate how a more mature understanding of this class of molecules could be driving the next generation of applications.

## Abstract 2

Since their original development, aptamers have been heralded as possible and promising new therapeutic modalities. Here, we explore the state of the art of (XNA) aptamers, and the bottlenecks the field is currently tackling.

## Graphical abstract

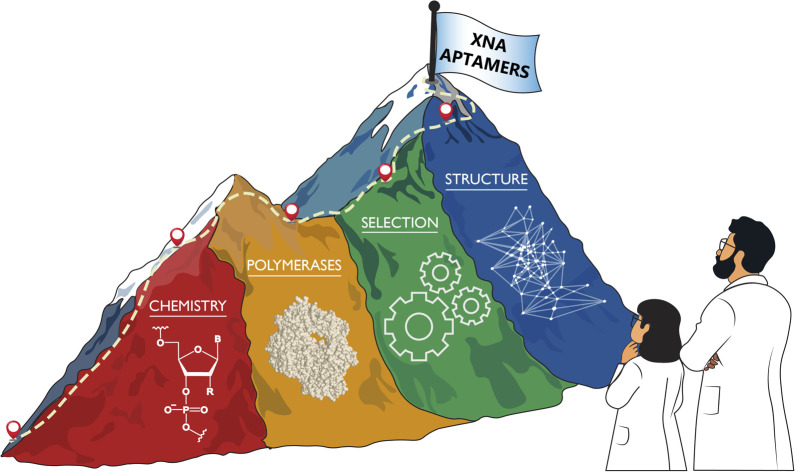

## Introduction

Aptamers are often defined as short single-stranded nucleic acids (*e.g.* DNA or RNA) that adopt stable three-dimensional structures capable of high-affinity and high-specificity binding to a target, which can include small organic compounds, peptides, proteins, other nucleic acids,^1,2^ and even whole cells.^3^ They are generally selected *in vitro* through an iterative process called systematic evolution of ligands by exponential enrichment (SELEX)^4^ or variations thereof.

Like antibodies, aptamers are highly versatile molecules that enable a wide range of applications including diagnostics (*e.g.* facilitating rapid and sensitive detection of disease markers, pathogens, and toxins^5^), biosensors (*e.g.* monitor dynamic changes in analyte concentrations, providing real-time information in clinical and environmental settings^6^) and therapeutics (*e.g.* Macugen and Izervay targeting neovascular wet age-related macular degeneration and geographic atrophy secondary to age-related macular degeneration, respectively^7,8^).

Nonetheless, the narrow chemical and biological stability of natural DNA and RNA represents a significant development barrier for aptamer applications, and a major one for therapeutic aptamer development. It can also be argued that the small chemical diversity among natural nucleobases and the structural rigidity of the (deoxy)ribose and phosphate backbones may further limit the potential structures (and therefore functions) that can be isolated.

Chemical modification of nucleic acids is possible and widely used,^9^ as universally accepted in the field of therapeutic antisense oligonucleotides (ASOs). While some of the groundwork for nucleotide modifications dates back to the 1960's^10–12^ – including the discovery of 2′-fluoro-(2′-F), 2′-*O*-methyl-(2′-OMe) and phosphate-backbone modifications – the first generation oligonucleotide modifications appeared decades later with therapeutic ASOs to equip them with inherent nuclease resistance: in 1986, the antiviral effect of a methylphosphonate oligonucleotide was reported,^13^ swiftly followed by the more successfully adopted and widely studied phosphorothioate (PS) modification in 1987.^14^ The progress made in the antisense oligonucleotide field allowed for the rapid introduction of chemical modifications to aptamers after their emergence in the 1990s,^4,5^ with the earliest publications appearing in 1994, incorporating 5-(1-pentynyl)-2′-deoxyuridine modifications,^15^ and in 1995, applying 2′-amino-2′-deoxypyrimidine and 2′-OMe modifications.^16,17^ Historically, 2′-F and 2′-OMe modifications are amongst the most popular aptamer chemical modifications, exemplified by Macugen and Izervay (relying on those 2′-sugar modifications along with conjugation with biocompatible polymers – *e.g.* PEG or cholesterol and 3′-end capping).

Whereas those initial modifications are still widely used in therapeutic oligonucleotides, the available toolkit has grown rapidly to include chemical modifications of nucleobase, sugar, phosphate and combinations thereof. Modifications can increase nuclease resistance, such as phosphorothioates^18^ and HNA,^19^ or introduce novel chemical moieties that alter the chemical properties of the molecule, such as the neutral backbone of phNA^20^ and PNA,^21^ or the hydrophobic moieties used in SOMAmers.^22^ Almost invariably, they also introduce changes to the sequence-function landscape that make mapping between landscapes impossible – that is, a *bona fide* aptamer in one nucleic acid chemistry does not necessarily remain an aptamer once the chemistry in changed.

**Fig. 1:**
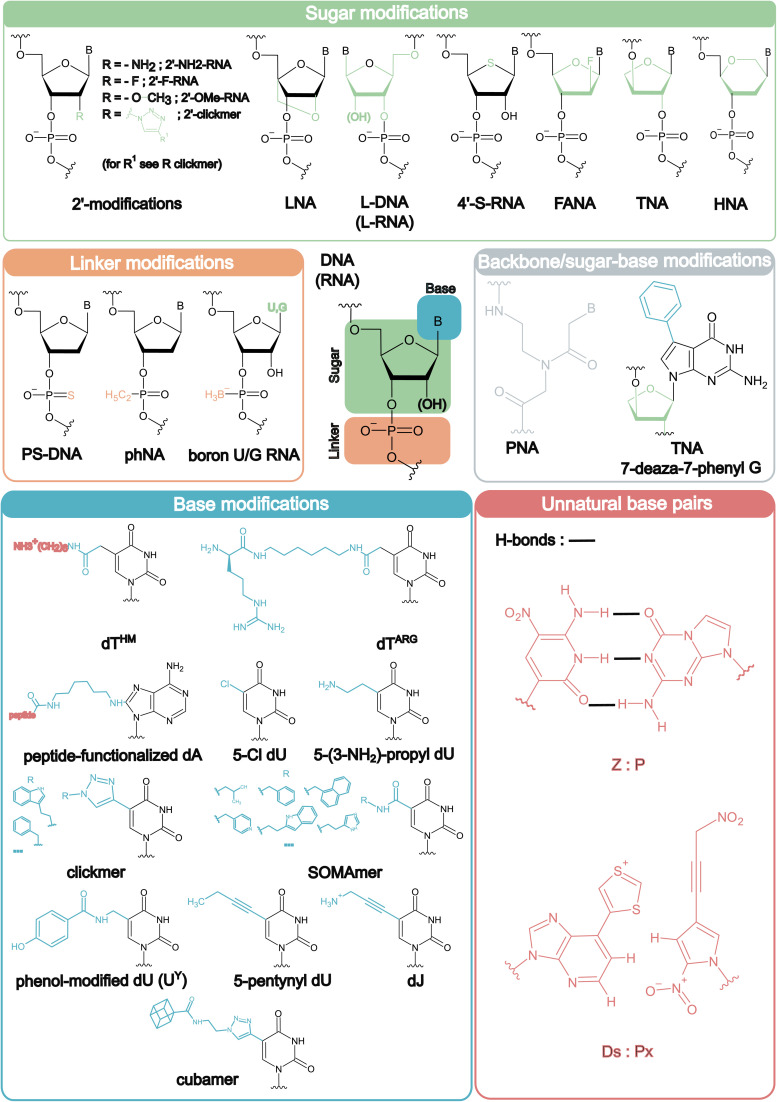
Structures of XNA chemistries mentioned in this review. Chemical structures are categorized according to the position of the modification (*i.e.* sugar, linker, base modifications or combinations thereof and unnatural base pairs), with the exact chemical groups different from their natural counterparts highlighted in the colour corresponding to their type of modification. For clickmers and SOMAmers the most incorporated R-groups are given. For Ds aptamers, both the Ds and Px bases are depicted.

The term ‘XNA’ (xenobiotic nucleic acids, also known as synthetic nucleic acids) was coined in literature in 2009 referring to sugar modifications.^23^ However, since then, XNA has been used more broadly for chemically modified nucleic acids for any sugar and/or backbone modification.^24^ In 2019, a new classification system was proposed, using XNA, DZA and DNY according to the position of the modification.^24^ While this distinction is important to consider (because of their different chemical and structural impacts), for the purpose of this review, we discuss XNA in its broadest term, concentrating on backbone and nucleobase modifications most relevant for aptamer development. Chemical structures (and the position of modification compared to the natural nucleotides) of the XNA chemistries discussed in this review are depicted in Fig. 1.

Selection of aptamers directly in XNA is increasingly possible, but development lags compared to RNA and DNA. Here, we review critically the current state of the field of XNA aptamers and highlight some of the persistent bottlenecks that affect not only XNA aptamers but the aptamer field in general. The aptamer field is extensive, and we apologize to our colleagues whose work we cannot include in this brief review. Other excellent reviews on aptamers,^3,25–29^ chemical modification of nucleic acids and XNAs,^9,30–32^ and *in silico* tools for aptamer development^33–36^ are available elsewhere.

## A brief history of aptamers

### The 1990's – first examples

The development of SELEX is a watershed moment for nucleic acid aptamers, but it did not emerge in a vacuum. As highlighted by Joyce in 1989 (ref. 37), all tools required to carry out the directed evolution of nucleic acids *in vitro* were available. Less than a year later, two groups published groundbreaking results reporting the first aptamers – Tuerk and Gold isolating the first RNA aptamer against the T4 DNA polymerase,^4^ and Ellington and Szostak isolating ligands against small molecules.^5^

**Fig. 2:**
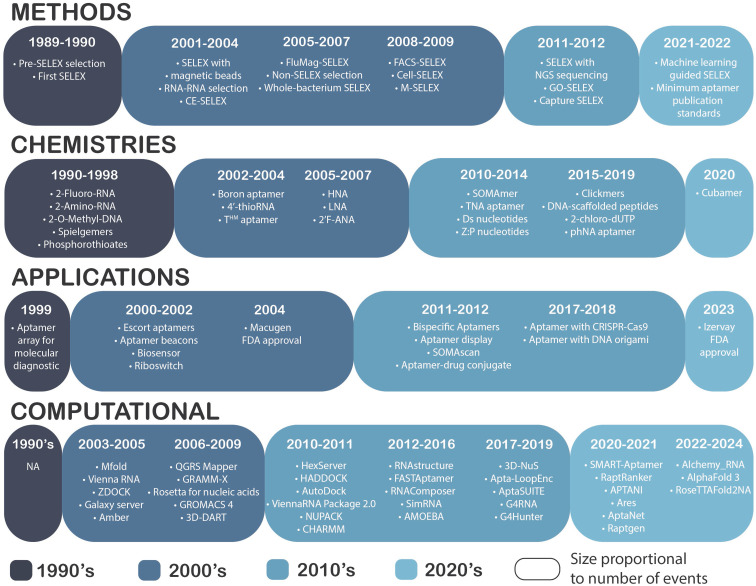
Key advancements in the aptamer field. To simplify the historical analysis of aptamers, we divided the field into four main domains: methods, chemistries, applications, and computational tools. Within the methods category, we primarily focus on advancements in aptamer selection techniques. In chemistries, we highlight incorporation of XNA chemistries into aptamers. Applications cover the integration of aptamers into new technologies, their combination with other methodologies, and the approval of aptamer-based medical products. Lastly, the computational category lists the development of *in silico* tools used for aptamer discovery, refinement, and understanding. In our figure, each decade is represented with a distinct color, and the size of each balloon within categories reflects the quantity of discoveries made in that decade.

The first decade in the aptamer field laid a solid foundation for subsequent decades of aptamer research (Fig. 2). Initially, the field quickly diversified its targets through the isolation of aptamers selected against various proteins, such as the thrombin-binding aptamer,^38^ and small molecules like ATP.^39^ Concurrently, significant technical advancements in SELEX methodologies further propelled this growth. By 1995, researchers had already isolated 49 aptamers, demonstrating a wide range of affinities spanning from 30 pM to 12 mM.^40^ Early research on aptamers also included enhancing aptamer properties through increasing their chemical diversity. Notably, some of these aptamers already incorporated chemically modified nucleosides like 2′-F, 2′-NH_2_,^40^ 2′-OMe^17^ and PS,^41^ and mirror images of RNA molecules had been developed.^42,43^

Elucidating the structures of aptamers was of interest for the field from its very beginning. By 1997, several aptamer structures had been elucidated through NMR spectroscopy, such as the RNA aptamer complexes binding adenosine monophosphate, flavin mononucleotide, arginine/citrulline, and tobramycin.^44^

Reaching the end of the decade, the first applications of aptamer technology emerged with aptamers being integrated into molecular diagnostic arrays.^45^

### The 2000's – focus on selection

The field of aptamer technology expanded significantly in the new millennium, driven by theoretical advancements and the proposal of aptamers as therapeutic and diagnostic agents.^46^ Methodologically, the early 2000s saw substantial progress with the integration of pivotal SELEX technologies such as magnetic beads^47^ and fluorescently labelled nucleic acid strands.^48^ Innovations like capillary electrophoresis-SELEX (CE-SELEX),^49^ FACS-SELEX using cell sorting,^50^ and Microfluidics SELEX (M-SELEX)^51^ integrated with micromagnetic principles expanded the capabilities of aptamer selection methods. The era also witnessed the advent of the first automated aptamer discovery and the introduction of non-SELEX methodologies.^52^

Advancements in aptamer selection methodologies enabled researchers to develop aptamers for new classes of targets, including whole cells and RNA. Cell-SELEX was successfully demonstrated through the isolation of aptamers against pathogenic bacteria (*e.g. Mycobacterium tuberculosis*^53^), cancer cells (*e.g.* liver cancer-specific aptamers^54^) and viral RNA domains (*e.g.* aptamers against Hepatitis C virus^55^). Moreover, the integration of Cell-SELEX with other novel selection methodologies facilitated the development of *in vivo* SELEX.^56^

The growth in aptamer technology paved the way for novel practical applications. The development of ‘escort aptamers’ enabled their use in *in vivo* diagnostics and treatments, marking a first contribution of aptamers to precision medicine.^57^ Additionally, aptamer beacons allowed direct, fast and sensitive protein detection.^58^ Beyond diagnostics, natural aptamers were also identified and adopted for genetic regulation through riboswitches, modulating gene expression by binding metabolites.^59^ The introduction of aptamer biosensors marked the first step in a leading application in the field.^60^

Chemical diversity in aptamer design flourished during this decade with the development of novel chemistries such as boron-containing aptamers,^61^ hexitol nucleic acid-containing aptamers (HNA),^62^ 4′-thioRNA,^63^ locked nucleic acids (LNA),^64^ 2′-fluoro-d-arabino-RNA (FANA),^65^ cationic-charged modified DNA aptamers (*e.g.* T^HM^),^66^ and the first combinations of modified chemistries, such as LNA with 2′-OMe.^67^ These advancements culminated in a pivotal moment with the FDA approval of Macugen in 2004 for the treatment of neovascular (wet) age-related macular degeneration,^68^ validating aptamers as viable therapeutic agents (while also establishing viable manufacturing and formulation strategies for aptamers) and highlighting their potential in biomedical research.

The 2000s also witnessed the emergence of computational tools essential for advancing aptamer research. Tools like Mfold^69^ and ViennaRNA^70^ provided the first predictions of RNA secondary structures critical for a better understanding of aptamer folding and stability. QGRS Mapper facilitated the identification of G-quadruplex structures within aptamers, enhancing their structural characterization.^71^ The era also saw the first pipeline for the prediction and visualization of 3D aptamer structures with 3D-DART.^72^ Moreover, during these years the first version of the Galaxy Platform was developed.^73^

Rigid docking methods employed by tools like ZDOCK^74^ and GRAMM-X^75^ facilitated exploration of aptamer–target binding surfaces, while flexible docking approaches emerged with tools like Rosetta, originally developed for proteins in 2004 (ref. 76) and later applied to nucleic acids in 2007.^77^ Molecular dynamics simulations with AMBER^78^ and GROMACS 4 (ref. 79) enabled researchers to simulate the dynamic behaviour of aptamer–target complexes, offering deeper insights into their stability and functional mechanisms. These computational tools laid the foundation for future *in silico* tools.

### The 2010's – integrating new technologies

In the following decade, additional SELEX methodologies were developed, now focused on overcoming previously described limitations of the process. Techniques like graphene oxide-assisted SELEX (GO-SELEX, utilizing graphene oxide for non-specific adsorption of ssDNA)^80^ and Capture SELEX (employing nucleic acid library immobilization on magnetic beads),^81^ emerged to bypass target immobilization prior to selection. The integration of Next-Generation Sequencing (NGS) in SELEX methodologies represented a paradigm shift, offering an alternative to traditional Sanger sequencing.^82^ This innovation led to the development of analytical tools such as AptaPLEX,^83^ AptaMotif,^84^ AptaCluster,^85^ AptaMut,^86^ and AptaTRACE,^87^ culminating in their combination into the AptaSUITE platform.^88^ AptaSUITE enables researchers to demultiplex NGS data, identify sequence motifs, cluster sequences based on locality-sensitive hashing (LSH), predict mutant affinity, and analyse structural enrichment of *k*-mers, becoming a cornerstone in contemporary aptamer NGS sequencing analysis.

Additional software tools for NGS data analysis emerged during this period, including FASTAptamer,^89^ facilitating sequence clustering based on Levenshtein edit distances, and APTANI,^90^ which leverages structural enrichment of *k*-mers and secondary structure information for aptamer identification. The update of Galaxy in 2016 provided an improved versatile web server platform for comprehensive nucleic-acid sequence analysis.^91^

Tools for predicting 2D structures, such as ViennaRNA package 2.0,^92^ NUPACK,^93^ and RNAstructure,^94^ were complemented by specialized software for G-quadruplex prediction like G4RNA^95^ and G4hunter.^96^ In 3D structure modelling, tools like RNAComposer,^97^ SimRNA,^98^ and 3D-Nus^99^ enhanced predictions despite their limitations in accuracy.

Advancements in predicting aptamer–target binding surfaces were achieved through tools like Hexserver^100^ for rigid docking and HADDOCK,^101^ AutoDock,^102^ and AutoDock Vina^103^ for flexible docking. Molecular dynamics simulations using CHARMM^104^ and AMOEBA^105^ were first used to provide insights into the dynamic behaviour of aptamer–target complexes. Machine learning applications also gained traction, exemplified by Apta-LoopEnc, which uses Support Vector Machines (SVM) to predict aptamer sequences based on their binding affinity using SELEX data from high and low affinity cycles.^106^

The 2010s also witnessed a significant growth in combining aptamers with diverse technologies. Innovations included bispecific aptamers,^107^ aptamer–drug conjugates^108^ for targeted drug delivery, integration of aptamers with DNA origami^109^ for enhanced structural interactions, and aptamer-functionalized lipopolymer delivery systems^110^ facilitating CRISPR-Cas9 plasmid transport for gene editing. Furthermore, advancements in aptamer display techniques were achieved through DNA nanostructure-decorated surfaces.^111^

The period witnessed new advancements in the chemical expansion of aptamers, such as the introduction of threose nucleic acids (TNA),^112^ and the incorporation of a genetically expanded alphabet using Ds,^113^ P and Z^114^ nucleobases. Additionally, there were innovations such as functionalized DNA structures like Clickmers^115^ and DNA-scaffolded peptides.^116^ Combinations of chemistries, such as TNA with 7-deaza-7-phenyl modified guanosines^117^ and 2′-OMe-DNA with HNA, were explored further.^118^

Significant milestones during this period include the development of the first aptamer with an uncharged backbone (phNA)^20^ and the creation of Slow Off-rate Modified Aptamers (SOMAmer),^119^ which later paved the way for the SOMAscan proteomics platform.^22^

Throughout the decade, clinical trials explored aptamer therapeutic potential across diverse medical conditions like macular degeneration, cancer, and diabetes. Some of the candidates did not progress past phase I or II, such as RB006, ARC1779, NU172, and ARC19499, aimed at addressing hemostasis; AS14, in the field of cancer treatment; and E10030 (Fovista™), for macular degeneration. Notably, other aptamers showed promise, such as NOX-A12 for cancer treatment, prompting TME Pharma to seek FDA Fast-Track Designation in February 2024. Similarly, NOX-E36 completed successful phase I and II trials for diabetes mellitus, indicating potential for further development as a treatment option.^120^

### The 2020's – possible watershed moment

In the current decade, computational tools aimed at advancing aptamer research have proliferated. These tools represent a shift from single-criterion strategies to multi-dimensional scoring methods, exemplified by SMART-Aptamer,^121^ RaptRanker,^122^ and APTANI2,^123^ offering more nuanced perspectives on aptamer sequence analysis.

Deep learning has propelled significant advancements in computational biology. Tools like AlphaFold 3 (ref. 124) and RoseTTAFold2NA^125^ showcase this progress, utilizing deep neural networks to predict protein and RNA structures with unprecedented accuracy. Similarly, innovations such as Alchemy_RNA2^126^ and Ares^127^ apply machine learning techniques to predict nucleic acid structures, leveraging evolutionary and biophysical data for improved insights.

This technological evolution parallels advancements in other areas of computational biology, such as AptaNet's aptamer–protein interactions prediction using machine learning,^128^ and generative aptamer design facilitated by Raptgen.^129^ Furthermore, the last few years also saw a milestone in aptamer discovery with the introduction of the first aptamer found using a machine learning-guided refinement and discovery protocol.^130^

Regarding novel aptamer chemistries, the field presented the integration of cubane moieties into aptamer structures^131^ – arguably combining the resulting improved stability with unique hydrogen bonding capabilities.

Clinical trials in the 2020s have underscored the diagnostic and therapeutic potential of aptamers across various applications. Diagnostic aptamers include the Saliva-based COVID-19 DNA Aptamer Test (NCT04974203) for rapid COVID-19 diagnosis, the Tenofovir (TFV) Aptamer-Based Biosensor (NCT04870671) for monitoring AIDS treatment adherence, molecular biosensors for the detection of bladder cancer (NCT02957370), and a labelled ssDNA aptamer for colorectal cancer diagnosis (NCT03385148).^120^ Therapeutic trials feature EYE001 (NCT00239928) for macular degeneration, NOX-H94 (NCT02079896) for anaemia of chronic disease, BT200 (NCT04677803) for hereditary bleeding disorders, and 68GA-Sgc8 (NCT03385148) against colorectal cancer. Notably, Izervay received FDA approval in 2023 for the treatment of geographic atrophy secondary to macular degeneration, marking a significant milestone in aptamer-based therapeutics.^120^

**Fig. 3:**
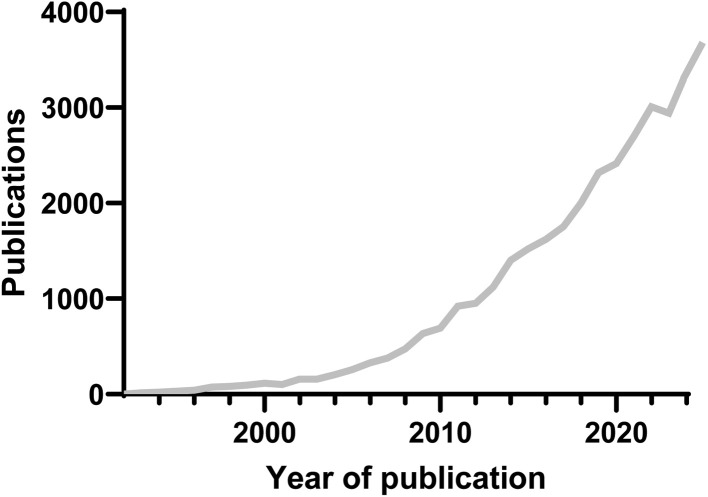
Publication analysis by year of publications containing the words “aptamer” or “aptamers” until December 31st, 2025 using the Web of Science (WoS) (https://mjl.clarivate.com/).

In 2022, the field achieved a pivotal standardization milestone with the publication of minimum aptamer publication standards,^132^ building upon initial suggestions from 2009.^133^ However, despite these advancements and the field's gain in popularity (Fig. 3), the literature still lacks comprehensive theoretical frameworks beyond traditional SELEX methodologies, highlighting an ongoing gap in aptamer theory despite the prolific practical and clinical advancements.

## Limits despite success

**Fig. 4:**
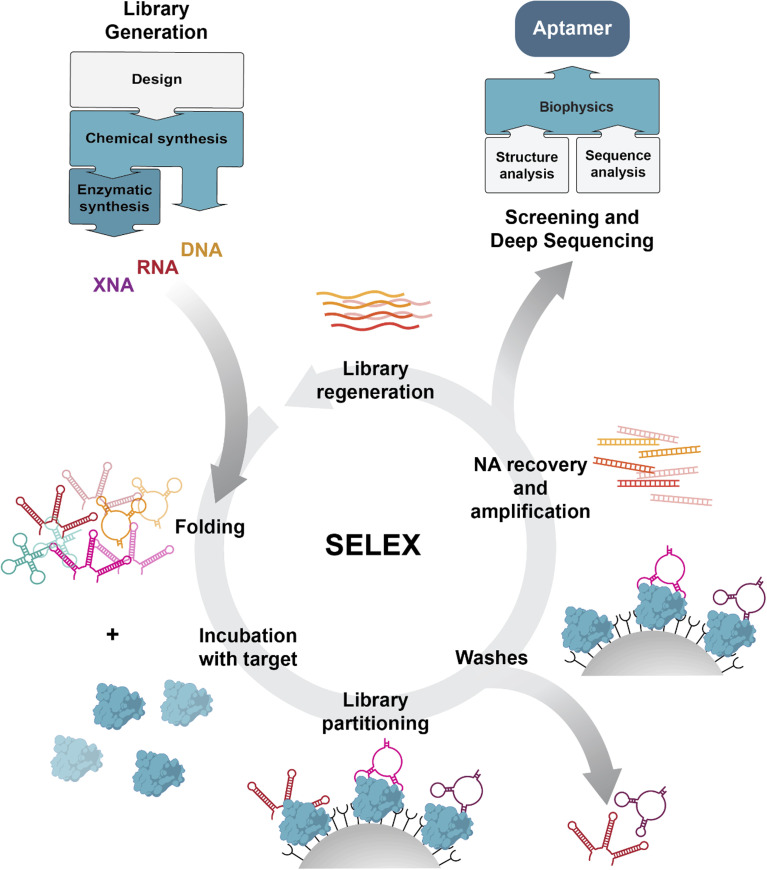
Overview of the aptamer selection process. SELEX consists of iterative rounds of library (re)generation, incubation with the target, partitioning and amplification of the retained sequences. However, depending on the chemistry used, the detailed SELEX process will look different. Library generation: the first step in the aptamer selection process is the library design and subsequent library generation. In most cases, a DNA library is first generated. For RNA or XNA chemistries, the DNA library is transcribed to either of them. For some XNA chemistries libraries are generated through direct chemical synthesis. Detailed reviews on considerations when designing your library can be found elsewhere.^136–139^ Main SELEX protocol: while the incubation with the target and partitioning steps are comparable between chemistries, subsequent amplification of binders and library regeneration require additional steps for RNA and XNA. To make amplifications possible, sequences need to be reverse transcribed into cDNA. After amplification these sequences are then transcribed back to the original chemistry. For RNA, established polymerases such as T7 RNA polymerase are used. For XNA, (reverse) transcriptases that can process these chemistries need to be available, incurring technological limitations. In some cases, *i.e.* with phosphorothioate, XNA can be directly amplified from the eluted sequences, resulting in shorter selection cycles. Various strategies to obtain ssXNA after PCR amplification exist but is here represented by the strategy utilizing magnetic beads and elution by denaturation. A common notable alternative to the depicted magnetic beads-based SELEX is Capture SELEX,^140^ in which the aptamer library is immobilized on magnetic beads instead of the target. Screening and deep sequencing: after the last selection round, the enriched sequences are determined and analysed using custom scripts, or through available platforms such as The Galaxy Project for sequence data handling, or AptaSUITE^141^ for further computational analyses.

Since its development, SELEX and its many variations have remained the gold standard in the field for the isolation of the highest affinity binders. Starting from a diverse library of random sequences, SELEX typically follows iterative cycles that can broadly be divided into four phases: (1) (re)generation of the library, (2) incubation with the target, (3) partitioning of binders from non-binders and (4) amplification of the retained sequences. The fundamentals of this selection process can be found in Fig. 4. Traditionally, stringency is increased between selection cycles – *e.g.* decreased target concentration, decreased incubation time or more elaborate washing steps – allowing the high-affinity binders within the library to outcompete the weaker ones. Once sufficient enrichment of high-affinity binders is observed, the remaining sequences are analyzed, and several aptamer-candidates are chosen for further characterization. In addition to affinity and specificity measurements, characterization can include stability tests (*e.g.* serum nuclease degradation^134^) and functionality descriptions (*e.g.* quantification of inhibitory effect^135^), with the goal of finding aptamer sequence(s) that satisfy the requirements of the selection. In a final stage, aptamers can also be fine-tuned, through truncations, chemical modifications, or conjugations.^28^

Research into aptamers has predominantly focused on addressing the inherent challenges associated with the selection process, *i.e.* SELEX modifications to optimize and streamline the SELEX protocol. Those SELEX modifications can encompass counter-selection steps (where similar targets are used in a negative selection round), alternative library designs (such as pre-structured libraries), or improved selection conditions, *e.g.* adaptations based on the intended application, promoting selection of the high-affinity binders or the addition of competitors. While those optimization strategies have increased the success rate in obtaining high-affinity aptamers against a diverse range of targets, SELEX still encounters both experimental challenges and theoretical limitations.^142^

On the practical front, SELEX remains a time- and labour-intensive procedure. Despite the emergence of innovative strategies aimed at enhancing or complementing SELEX, selection strategies continue (mostly) to function as black boxes, with few tools to monitor the progression of the selection campaign.^143^ In addition, the field lacks predictive tools to assess the likelihood of selection success before its execution.

When it comes to data analysis, the disclosure of analysis pipelines, software, or codes is infrequent. Moreover, there is a notable scarcity of detailed criteria for sequence selection and limited information on the number of sequences tested before arriving at a successful outcome. The field exhibits a significant survivor bias, and the absence of a repository for ‘difficult’ targets or unsuccessful selection attempts perpetuates a cycle of repeated mistakes among scientists across various laboratories. Nonetheless, in recent years, the field has become more aware of and concerned with the need for standardization, as exemplified by the publication of the minimum aptamer publication standards.^132^

Nonetheless, we believe that the field needs to go further, establishing a formal (and quantitatively robust) definition for aptamers. The current definition, which is qualitative, is ambiguous and leaves too much dependent on the context of the characterization, which often goes unreported. For instance, the reporting of a DNA sequence identical to a previously reported RNA aptamer against hen egg lysozyme:^144^ despite high affinities, compatible with aptamer definitions, the natural affinity of nucleic acids for a positively charged lysozyme protein, through electrostatic interactions, was not considered.^139^ In fact, much of the declined trust in the aptamer field stems, in our opinion, from the lack of guidelines and standards, mirroring the challenges faced by therapeutic antibodies due to overlooked specificity concerns.^145–147^

The problems caused by the lack of robust definition are exacerbated when chemical modifications are introduced or if different nucleic acid chemistries (*i.e.* XNAs) are used for aptamer development. In those cases, affinity alone is not enough to describe an aptamer, and specificity is not always clearly defined. Post-SELEX replacement of nucleic acid moieties with chemical analogues often compromises aptamer function^63,148,149^ but direct selection in XNA incurs greater technical challenges than those of SELEX alone.

## XNA aptamer selection

The complexity of the selection process increases when chemistries other than the traditional ones are used. XNA modifications can be introduced to aptamers either post-SELEX, or through adjusted SELEX protocols (Table 1). In the first method, researchers systematically introduce modifications to a previously selected aptamer and a number of modified aptamers are analyzed to evaluate their affinity, selectivity, and stability. Although this method is widely used in the field,^8,67,148,150–153^ it is time-consuming and labour-intensive due to the unknown role and impact a given XNA substitution could have on aptamer folding and function. Furthermore, due to the usual trade-off between affinity and aptamer chemical modification it is rare to identify fully-substituted aptamers this way.^3,63,148^

**Table 1:** An overview of XNA chemistries already reported in aptamers: their representative affinity, specificity and SELEX tools. The overview is extensive but not thorough as we emphasise the diversity of XNA chemistries successfully used in aptamer selections. For each chemistry, the first reported aptamer is summarised. In cases where multiple aptamers were co-reported we include the specific aptamer we used for the table or the range of affinities for the isolated aptamers. Chemistries highlighted in 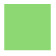 (green) have been incorporated during selection, 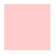 (pink) only after selection and 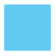 (blue) both during and after selection. Where reported, targets screened for specificity are noted. No off-target binding was reported (unless otherwise stated in the table). The polymerases used in the SELEX process are listed with polymerases used directly for the XNA chemistry shown in green (if commercially available) or red (if not commercially available). N.R. – not reported. N.A. not applicable

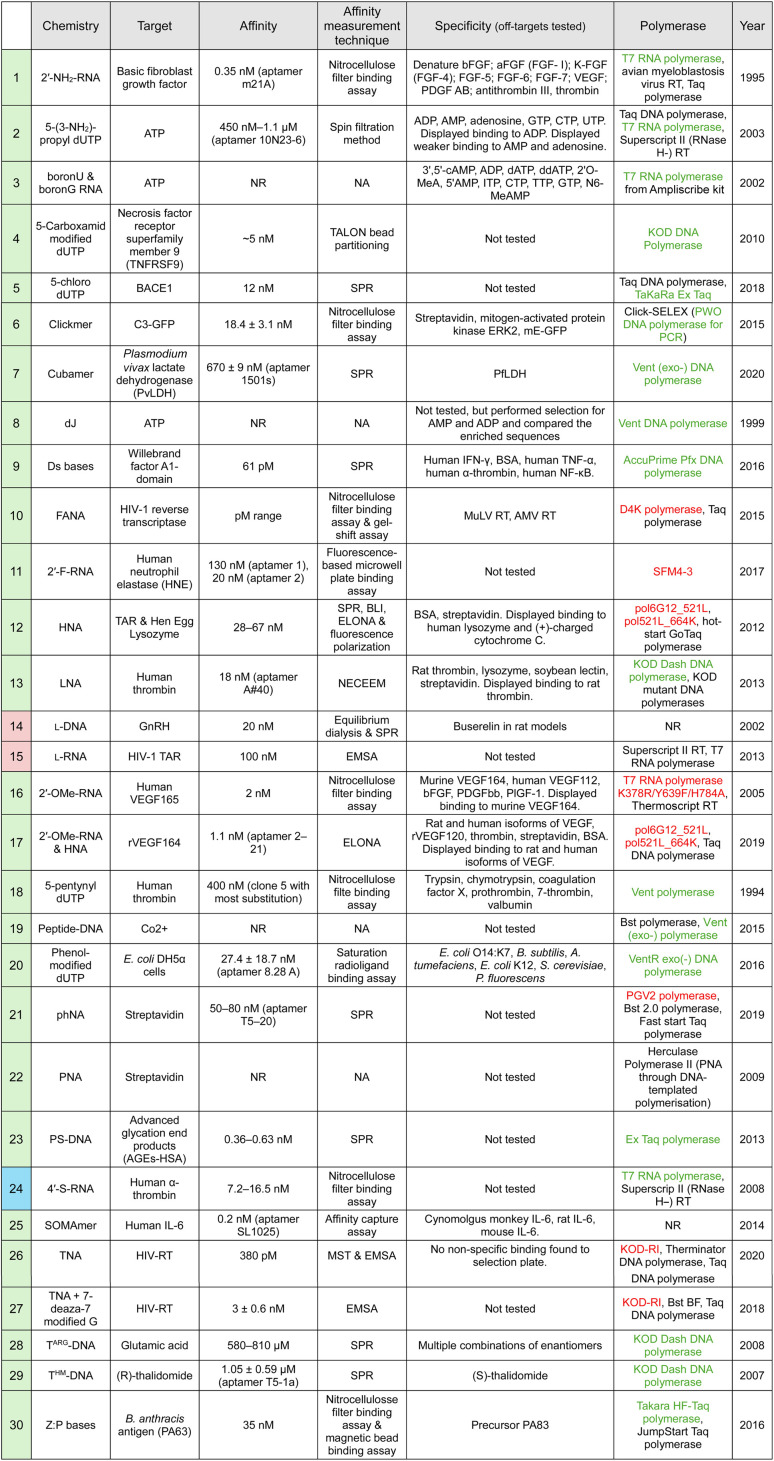

The alternative solution is to adapt the selection protocol to directly integrate XNA chemistries. However, this requires a re-evaluation of every step in the SELEX process – from library generation to selection cycles – to ensure optimal efficiency and success.

Creating libraries for DNA is relatively straightforward, often involving the purchase of pre-synthesized libraries from specialized companies. For RNA libraries, a common practice involves transcribing DNA libraries, facilitated by commercially available kits. However, with XNA, building blocks are not often commercially available (thus requiring specialist chemistry for synthesis), chemical synthesis of libraries is often not possible (because of lower chemical couplings of the building blocks), and synthesis of the nucleotide triphosphate equivalent is not as efficient as for natural (deoxy)ribonucleotides.

Consequently, XNA SELEX adds two extra requirements: polymerases that can synthesize XNA libraries for selection and enzymes that can access the XNA information in the library post-selection. While some base and sugar modifications can be efficiently incorporated by natural or commercially available engineered polymerase variants, most backbone-modified XNAs rely on specialized enzymes that are not widely available (see next section).

Other steps in the SELEX process are also affected. While conventional DNA-based SELEX protocols can rely on enzymes like lambda exonuclease to degrade 5′-phosphorylated template strands in double-stranded DNA to obtain a library of single strands, XNA-based SELEX often requires alternatives, *e.g.* isolation through PAGE,^154,155^ degradation of the DNA templates through DNase I,^156^ magnetic beads^155,157^ or other innovative solutions^158,159^ – invariably increasing the duration and cost of SELEX.

Additionally, while the field has made great progress since the turn of the century in terms of computational analyses of aptamer selection and 2D/3D structure predictions, knowledge of XNA structures is very limited, curtailing possible structure prediction efforts.

### Polymerases as a bottleneck for XNA aptamer discovery

The existence of a given XNA chemistry, and even the demonstration that it can be processed by an engineered polymerase, does not necessarily imply that its full sequence landscape (and therefore functional landscape) is experimentally accessible. Efficient SELEX requires polymerases capable of efficient synthesis (XNA from DNA templates and DNA from XNA templates) in reaction conditions that are often challenging, *e.g.* biased sequences during library synthesis, or low template concentrations after NA recovery.

XNA synthesis is constrained by the stability of the synthesised duplex, and by the polymerase's sequence tolerance and error profile. The reported tPhoNA (phosphonomethyl-threosyl nucleic acid) showed significant stalling when two Ts were incorporated sequentially.^160^ Similarly, early TNA polymerases showed significant G:G mispairing.^161^ In both cases, the result would be the selective exclusion of certain sequences from the libraries under selection. Such constraints have long been recognised in nucleic acid evolution studies, where the success of selection experiments depends on how amenable to replication a sequence is, as much as on its binding capability.^162,163^

For DNA selections, polymerase performance has historically been a decisive enabling factor. Thermostable DNA polymerases, particularly Taq and its derivatives, allow detection and amplification from extremely small amounts of template, while maintaining sufficient fidelity to propagate functional sequences across selection cycles.^164^ The ability to amplify minute amounts of template directly, affects selection stringency: higher stringency conditions recover fewer nucleic acid molecules, and only robust amplification permits those stringent conditions to be applied without losing the evolving population.^165^ Moreover, DNA PCR amplification bias has been extensively studied, enabling researchers to mitigate its impact during SELEX and preserve a substantial fraction of library diversity, even for structured templates.^166,167^

A similar principle applies to RNA selections, which typically include transcription, reverse transcription, and PCR amplification steps. Reverse transcriptases such as M-MLV RT, AMV RT, and thermostable variants (*e.g.* SuperScript derivatives) are routinely used to convert selected RNA into cDNA prior to PCR.^165,168^ Their efficiency is sufficiently high that even structured RNAs can often be recovered and propagated.

For XNA chemistries that structurally resemble DNA/RNA (*e.g.* most modifications on the 2′ position or the nucleobase itself) or where modification of all four bases is not coveted, natural polymerases or commercially available engineered ones can still perform adequately (*e.g.* Therminator™ DNA polymerase incorporating TNA^112^). Nucleobase modifications can often be tolerated by natural polymerases and therefore can be directly amplified^157,169–171^ or amplified in their reverse transcription or transcription steps.^172–174^ Sugar or backbone modifications perform less well, *e.g.* PS, which can be directly amplified using Taq polymerase.^175,176^

For more divergent XNA chemistries, engineered XNA polymerases are essential and, despite advances in the field, remain a significant practical constraint. Consequently, their use for *in vitro* selection of XNA aptamers remains rare. In 2005, probably the first report of fully substituted XNA aptamer selections, Burmeister *et al.* used a T7 triple-mutant Y639F/H784A/K378R for the selection of a fully modified 2′-OMe aptamer binding VEGF.^177^ Significant advances in DNA polymerase engineering have enabled new families of XNA polymerases to be developed, and with that the expansion of the available repertoire of XNAs accessible to SELEX.^19^ The first report of *in vitro* selected HNA (1,5-anhydrohexitol nucleic acids) aptamers against HIV-1 TAR and hen egg lysozyme was quickly followed by multiple reports covering a wide range of nucleic acid chemistries, summarized in Table 1.

At present, most XNA polymerases derive from archaeal hyperthermophilic B-family DNA polymerases.^178–181^ Although bearing lower processivity than equivalent A-family polymerases, B-family enzymes generally exhibit greater substrate tolerance, making them suitable starting points for XNA polymerase engineering. A wide range of selected and engineered polymerases are available (both for XNA synthesis and XNA reverse transcription),^19,182–184^ but comparison of their biochemical activity highlights common engineering trade-offs.^178^ Mutations that enable XNA synthesis typically expand the substrate spectrum of the enzymes (rather than create polymerases specialist on the new chemistry) and do not remove their DNA polymerase activity. This broader substrate spectrum is often linked to decrease fidelity (beyond mutations that simply inactivate the error proofing mechanisms).^3,27,185^ Similar XNA chemistry variation is also observed among the engineered transcriptases.

In the context of SELEX, those features are not neutral. Lower amplification efficiencies and lower fidelity, limit the stringency that can be applied in a SELEX round, favour amplification of sequences that are easier to replicate, and selectively bias SELEX towards areas of the sequence space that are less rugged. As such, enrichment reflects not only function, but also the compatibility of the sequences with the XNA chemistry and polymerases being used. This process is exacerbated in XNA chemistries where exponential amplification is not possible, *i.e.* systems where XNA synthesis (or RT) relies on a transcription-like linear amplification or in direct synthesis of XNA from a DNA template – processes that limit sequence enrichment.^178,186^

There are also additional consequences to the SELEX process. Classical SELEX typically requires approximately 8–15 selection rounds before enrichment is achieved.^5,165^ Each selection round creates a new opportunity for polymerase-mediated effects to emerge, such as biases, parasites and amplification artefacts. Indeed, the accumulation of PCR byproducts, amplification parasites and artefacts is well documented even in SELEX for natural nucleic acids.^166,187^

### Factors impacting XNA diversity and adoption

While we highlight representatives from a range of XNA chemistries in Table 1, that does not reflect the unequal frequency of XNA chemistries in the aptamer literature, which is shaped by feasibility rather than theoretical chemical potential. Multiple factors play into which chemistries dominate in the research field: (1) the time a chemistry has had to mature, (2) selection compatibility (both enzymatic and chemical synthesis), (3) a balance between benefit *vs.* cost-effectiveness and (4) the extent to which the XNA has been characterised (*e.g.* reached clinical trials). That is summarised in Table 2.

**Table 2:** Frequency of XNA chemistries in the literature. The XNA chemistries discussed in this review are divided into three categories: most frequently used chemistries (more than 100 reports); moderately represented chemistries (between 5 and 100 reports) and rare chemistries (up to 5 reports); based on how many times they appear in the literature. Although SOMAmers appear a moderate number of times, they have been categorised as a frequently used chemistry due to the commercialized SOMAscan platform (now part of Illumina's proteomics portfolio) which is known to have in excess of two thousand aptamers. Factors that influence XNA chemistry adoption are highlighted for each chemistry: 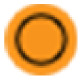 – good compatibility with selection (efficient enzymatic/chemical synthesis), 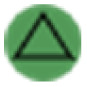 – improved biological or chemical stability, 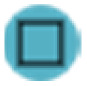 – synthetic nucleotide triphosphates are commercially available, 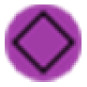 – known pharmacokinetic properties, 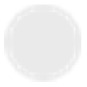 – not known or not applicable. For the category of rare chemistries, XNAs are subdivided into those researched for their improved functionality (*e.g.* 4′-S-RNA increases nuclease resistance; cubamer adds cationic side chains) and those that use XNA aptamer selection as a proof of principle (often to demonstrate polymerase capabilities)

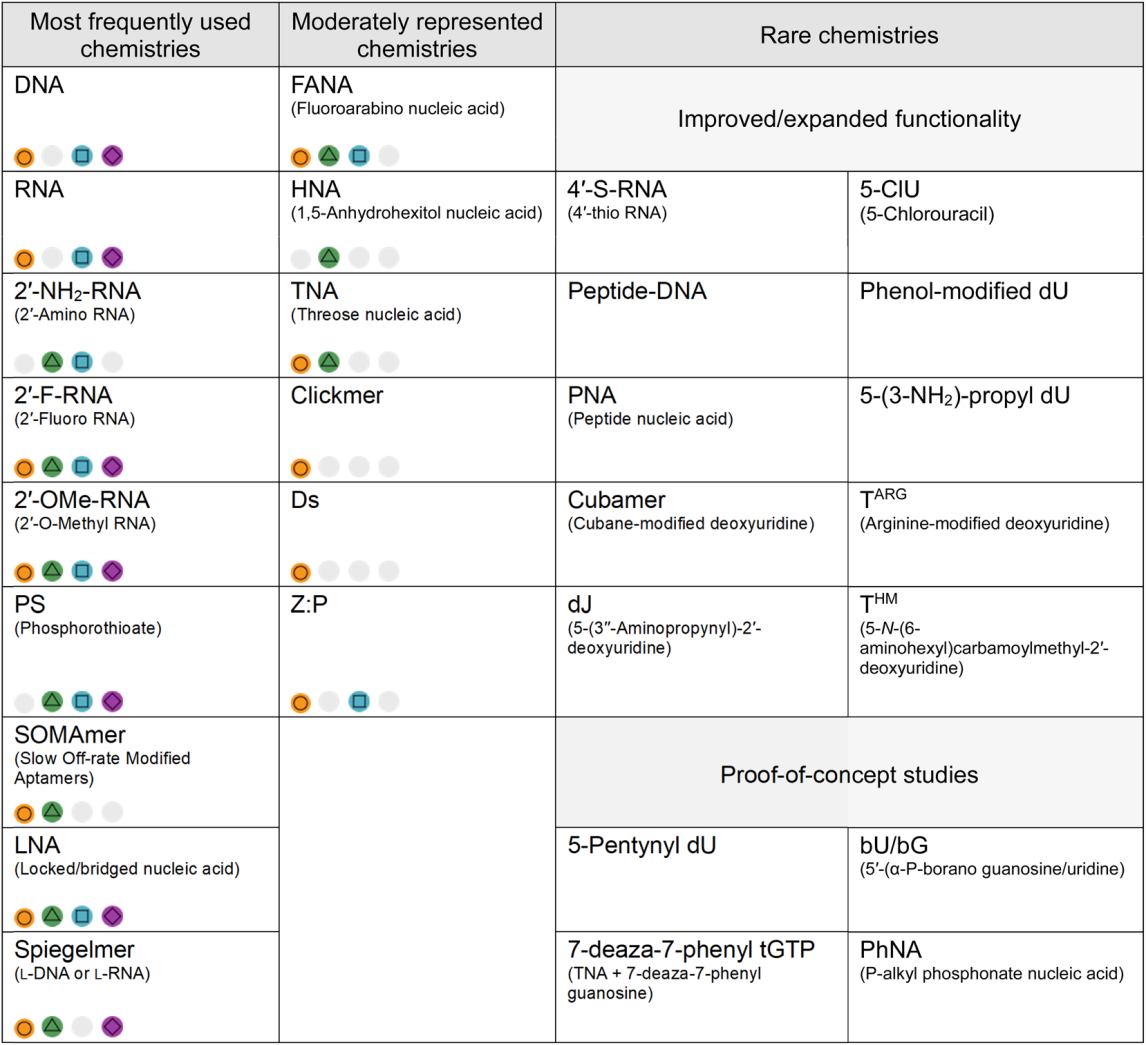

Firstly, modifications appearing earlier in the literature tend to still be more heavily featured in XNA aptamers to date. Modifications such as 2′-F and 2′-OMe were developed decades ago, in 1961 (ref. 12) and 1965,^11^ respectively, and were incorporated into aptamers shortly after the emergence of the research field, primarily as strategies to enhance nuclease resistance. By the time one of the earliest mentions of FANA aptamers appeared in literature in 2007,^65^ 2′-F/OMe modifications already had accumulated more than a decade of substantial methodological refinement and empirical validation. Similarly, frequently reported sugar modifications such as LNA^67,148^ already appear in literature as post-SELEX modified aptamers before the 2010's – preceding the use of specialised engineered polymerases. In comparison, chemistries such as HNA and TNA for which the tools have only been reported in 2012 and where optimization is still ongoing, lag behind the field.^19,112,188,189^

Secondly, as discussed above, polymerases play a crucial role in SELEX and it thus contributes substantially to the disparity among XNA chemistries. Unsurprisingly, the prevalence of reported XNA aptamers mirrors the availability of robust polymerase systems. The most frequently used chemistries (each reported in more than 100 aptamers) include 2′-F/OMe,^8,17,177,190–194^ SOMAmers,^174,193,195^ LNA,^67,148,156,158,196,197^ spiegelmers,^198–203^ and PS,^18,135,176,204–207^ have established SELEX pipelines and can be amplified using commercially available or robust engineered polymerases. In contrast, chemistries such as HNA remain less frequently reported despite favourable properties (including strong nuclease resistance, serum stability and duplex thermostability). HNA-based selections have yielded high-affinity ligands, even achieving low-nanomolar binding constants when combined with 2′-OMe-modified bases.^118^ Similarly, TNA aptamers have reached low-nanomolar affinities following the development of a robust KOD-RI polymerase.^117,154,184,208,209^ Nevertheless, because both platforms rely on specialized engineered polymerases and tailored synthetic methodologies, their broader implementation remains limited.

Accessibility to XNA precursors also affects which XNAs are used. Many heavily modified XNAs – such as some 5′-substituted dUTPs,^210–212^ Cubamer,^157^ dJ,^213^ phosphonate backbones,^214^ T^ARG^,^172^ and T^HM^^66,173^ – require bespoke synthetic routes, appear infrequently in the literature and are limited to a few specialist research groups.

Equally, solid-phase synthesis of selected sequences should be accessible. While polymerases are used during selection rounds, solid-phase synthesis (chemical synthesis of oligonucleotides using phosphoramidite chemistry) is usually necessary to create sequences for further characterisation or production of the selected aptamer. At that stage, the efficiency of the solid-phase coupling and the cost-effectiveness of the process become relevant bottlenecks. For example, while both 2′-F and 2′-NH_2_ modifications are relatively easily synthesised enzymatically, 2′-F quickly became preferred over 2′-NH_2_ because of higher coupling efficiency and additional deprotection steps required during purification.^215^

Last, is the potential for a given XNA to provide improved functionality, whether by providing greater chemical (or biological) stability, or by introducing greater chemical diversity to the oligomer (thus allowing a wider repertoire of intra- and inter-molecular interactions). For example, 2′-F/OMe modifications are routinely added to RNA aptamers as they improve endonuclease resistance due to the 2′ modification replacing the required 2′-hydroxyl group. SOMAmers and clickmers^216–219^ both expand functional diversity by incorporating hydrophobic or aromatic side chains – mimicking amino acid side chains – while simultaneously enhancing stability and, in many cases, increase binding affinity.^119,174^ Their translation into commercial platforms illustrates the importance of accessible standard protocols and sought-after novel interactions.

Adoption of certain chemistries in therapeutic contexts is limited by our understanding of their pharmacokinetic behaviour. Only a small number of aptamer chemistries have progressed to clinical trials (DNA, RNA, 2′-F/OMe, spiegelmers) and consequently their pharmacokinetic properties are comparatively well characterized.^8,190,220^ Many other modifications have only been tested in *in vivo* mouse or rat models. For instance, SOMAmers demonstrated slower plasma clearance (dependent on the number and nature of modifications) in rat models.^221^ For other XNA chemistries, pharmacokinetic insights remain more limited and are often derived indirectly. Therapeutic antisense oligonucleotides have given insight into the pharmacokinetics of chemistries such as LNA and PS, which are expected to mirror aptamer behaviour.^222,223^ However, quantitative pharmacokinetic parameters for many newer or more obscure XNAs remain largely undefined.

## Antibodies *vs.* aptamers

Given the more than two decades of head start, it is not surprising that antibodies have established themselves as a dominant force in both the diagnostic and therapeutic markets. In the period from 2018 to 2023, regulatory agencies such as the FDA and EMA granted approvals for 109 monoclonal antibodies and only 16 nucleic acid-based therapeutics, with merely one belonging to the aptamer category. This underrepresentation of aptamers highlights not only the nascent state of the field but also the limited understanding of the therapeutic potential of aptamers.^224,225^

Determination of nucleic acid structures has historically been more difficult than that of proteins. This is reflected in the number of available structures in public databases, where proteins in the PDB database^226^ surpass 1.7 million structures, providing a comprehensive understanding of protein configurations, compared to little under 60 thousand for nucleic acids (35 426 for DNA and 19 635 for RNA) in the PDB and NDB databases. The lower number of available structures, the narrower chemical spectrum of nucleic acids, and our incomplete understanding of interactions beyond Watson–Crick base-pairing currently limit our progress on the *de novo* structure prediction and on the structure-based design of aptamers.

X-ray crystallography and NMR spectroscopy, each bearing their own advantages and challenges, are the common approaches for aptamer structure determination. High-quality crystals are difficult to isolate for nucleic acids and as a result, many of the available structures have been obtained through NMR. However, NMR is only suitable for smaller complexes (typically aptamers of 30–50 nucleotides and smaller proteins), due to narrow chemistry resulting in spectral overlap that emerges quickly with increasing number of nucleotides. Newer strategies, such as cryo-electron microscopy (cryo-EM), are gaining traction but unbound aptamer structure will remain difficult to determine (because of their natural structural plasticity).^227^

The accuracy of aptamer structure prediction tools, typically trained by the relatively limited known nucleic acid structures, lags significantly compared to protein structure prediction methods. At CASP15, prediction strategies such as those used by the Yang-Server team – trRosettaX2 (an improved version of trRosettaX^228^) and AlphaFold2 (ref. 229) – reached TM-scores of 0.876 with backbone accuracies of 0.96 Å RMSD for proteins. The latest release from DeepMind, AlphaFold 3, promises even higher accuracy for protein monomers and enhanced predictions for protein–protein interactions.^124^

In contrast, CASP15 marked the first inclusion of RNA structure prediction.^230^ Across 12 RNA targets of varying complexity – ranging from those with identifiable templates to those without – predictions exhibited RMSD values spanning 2 Å to nearly 17 Å. The field did not anticipate achieving atomic-level precision and the current emphasis remains on accurately reproducing overall folding shapes.^124^ Nonetheless, *in silico* approaches for nucleic acid structure determination are gathering pace (see next section).

A gap between antibodies and aptamers is also present in the diagnostic landscape, where the antibody-based diagnostic market, valued at US $20,000 million in 2017 and projected to reach US $35,000 million by 2026, reflects the widespread adoption and established role of antibody-based diagnostics in disease detection and monitoring.^231^ Despite the potential advantages of aptamers (*i.e.* small size, cheaper production with low batch-to-batch variability, better thermal stability for transport and storage) that would make them highly relevant for point-of-care testing,^232^ only 10 are undergoing clinical trials for the development of diagnostics.

Still, aptamer-based diagnostic technologies are entering the market:^233^ companies like NeoVentures (https://neoaptamers.com/), who provide diagnostics, detection kits and aptamer-based affinity columns; and Aptamer Science Inc. (https://aptsci.com/en/), who commercialize several biomarker detection kits. Another notable example is the collaboration between SomaLogic and Illumina, announced in 2022, which combines the SomaScan® Proteomics Assay with high throughput NGS platforms to support multi-omic profiling for novel biomarker discovery.

Finally, the two technologies are not incompatible and can be combined, *e.g.* with aptamer–antibody conjugates, where aptamer pharmacokinetic properties can be improved by the conjugation to the larger antibodies (or other proteins), deep tissue penetration can be enhanced, or their conjugation can work synergistically for a more potent inhibitory effect.^234^

### XNA aptamer structure determination through molecular modelling

As mentioned above, while progress is being made in the development of computational tools for aptamer structure prediction, the “AlphaFold” moment for the field has not happened yet and will require advancements in both computational algorithms and the availability of high-quality structural data for aptamers.^235^ Still, companies are already entering the market delivering custom DNA and RNA aptamers isolated *in silico* from AI-based platforms (*e.g.* Xelari, https://www.xelari.com).

The scarcity of experimental XNA aptamer structures may complicate the use of AI-based methods for structure prediction, but it does not preclude the exploration of alternative computational strategies. In this regard, molecular modelling offers an interesting complementary route, in line with integrative structure-modelling approaches recently proposed for RNA and protein-RNA complexes.^236^

Molecular modelling of XNA aptamers faces two major challenges: (1) accurate parametrisation of the XNA chemistry and (2) predicting the aptamer folding from scratch. Parametrisation requires deriving force field parameters that accurately describe the structural and energetic behaviour of the biomolecule, including both bonded and non-bonded interactions.^237,238^ While well-established force fields exist for DNA and RNA^239,240^ they are generally insufficient to accurately describe the diverse chemical modifications present in XNAs, requiring customised parameters.

Obtaining accurate XNA parameters typically involves quantum mechanical (QM) calculations to model the linker and (or) the nucleoside. Significant progress has been made in recent years, particularly in conformational sampling of five- and six-membered (sugar) rings.^241^ For instance, the pucker.rs toolkit^242^ simplifies the generation of coordinates required for QM-based conformational sampling of sugar rings and linkers. Representative structures obtained from such sampling can then be used for force field parametrisation. Bonded and non-bonded parameters are typically derived using tools such as ParamFit, while partial charges are determined separately *via* ESP/RESP calculations.^243–246^

This methodology has been successfully applied in several studies to parametrise diverse XNA oligonucleotides, including HNA, dXyNA, TNA and tPhoNA. Using the resulting force fields, molecular dynamics (MD) simulations were performed, and the predicted structures obtained were found to be in good agreement with available experimental data.^245,247^ These examples demonstrate that such computational pipelines are feasible and can yield reliable structural models for chemically modified oligonucleotides. Crucially, this approach could, in principle, also be extended to XNA aptamers. Most recently, AmberTools 2025 introduced the modXNA module,^248^ providing a framework for XNA parameterisation within AMBER and simplifying access to parameters for modified nucleotides. While current validations focus on relatively small modifications (*e.g.*, phosphorothioate, 2′F, 2′OMe, 5FU, methylated bases), it represents an encouraging step towards broader accessibility of XNA modelling tools.

The second challenge – predicting the folding of XNA aptamers – remains inherently more complex. Conventional MD simulations often struggle to explore the full folding landscape, as the system can become trapped in local energy minima. To overcome these limitations, enhanced sampling methods such as replica-exchange molecular dynamics (REMD) and simulated annealing, originally developed in the field of protein folding, have proven useful and can be applied to study aptamer folding.^249,250^ In REMD, several copies of the system are simulated simultaneously at different temperatures, occasionally swapping configurations to help it explore otherwise inaccessible states. Simulated annealing involves heating the system and then slowly cooling it, allowing it to sample a wider range of conformations. Simple restraints can guide the folding toward plausible structures, which are then refined in longer simulations to access stability. This strategy was successfully applied to predict the folded structure of an HNA aptamer, though its implementation remains challenging and often involves rounds of trial-and-error approaches.^251^

In parallel, for DNA and RNA aptamers, computational workflows have been developed to predict how an aptamer folds in complex with its target. These typically involve secondary structure prediction, 3D modelling, docking to the target, and subsequent MD simulations to evaluate stability and interactions.^33,35^ However, these workflows have so far been applied only to DNA and RNA, and extending them to XNA systems will require further development.

In the long term, as molecular modelling of XNA aptamers becomes increasingly accurate, the resulting structural ensembles could provide valuable training data for AI models. This would help alleviate the current scarcity of experimental structures and gradually bridge the gap between physics-based simulations and data-driven predictive approaches, though this remains a long-term goal.

## The future of (XNA) aptamers

The aptamer field has undergone significant evolution across its various branches since its beginning in the 90 s. Throughout much of that time, the focus in the field has been to enhance the efficiency and reproducibility of the aptamer discovery process.^252^ Early successes, targeting proteins with high pI like lysozyme^47,253–255^ and thrombin,^211,256–259^ may have contributed to the complexities in aptamer development being underestimated, which has led to the historical paradigm that aptamer isolation is contingent upon luck to encounter a high-affinity sequence in the initial nucleic acid population.^260–262^ Recent insights, however, highlight the importance of the starting population composition,^261,262^ which has been combined with computational tools to construct improved libraries.^263^

NGS has accelerated the identification of putative aptamers by enabling high-throughput analysis of nucleic acid populations, yet it introduces novel challenges, particularly in data interpretation.^264^ Traditional analysis methods predominantly focus on sequence-level information, often overlooking crucial structural attributes that govern aptamer functionality. Innovations in clustering algorithms designed to group aptamer sequences based on their structural profiles represent a fast-growing area of research,^264^ but their adoption in aptamer discovery pipelines is still emerging.

Lastly, the expanding repertoire of available XNA chemistries has direct consequences to the aptamer selection toolkit, offering unique properties that may enhance pharmacokinetic profiles or introduce novel chemical characteristics^198,199^ – but for which *in silico* tools are scarce.^265^

Nevertheless, increased effort without a change in paradigm will not be enough to bring aptamers into the limelight. From a more theoretical perspective, several critical aspects of the field demand attention.

First, there is a need for a more precise and objective definition of aptamers. The absence of a clear, universally accepted definition has led to ambiguity in distinguishing aptamers from single stranded nucleic acids with weak or nonspecific binding capabilities.^144,266^ In addition, there have been historical misconceptions in the field regarding the correlation between high affinity and high selectivity.^267^ It took nearly a decade for these assumptions to be challenged and revised.^268^ Therefore, there is a recognized need to establish a formal definition that comprehensively incorporates both affinity and selectivity in a paradigm that can be experimentally validated.

Second, although aptamers are often compared to antibodies, they are a unique class of biopolymers and require their own development paradigm. Aptamers are more complex than proteins, capable of reversible changes in response to environmental conditions,^269,270^ and exist within a very different sequence/functional landscape. In practical terms, it means that we have to rewrite our expectations on biophysical behaviour of aptamers and to develop tools that capture their true potential (or at least highlight their strength).

Third, the narrow chemical and biological stability of natural nucleic acids means that XNAs, irrespective of which nucleotide moiety is chemically modified, will become a central aspect of the future of therapeutic (and possibly also diagnostic) aptamers. The different XNA chemistries counteract the perceived chemical monotony of nucleic acids (*i.e.* the four natural nucleobases *versus* the 20 natural amino acids) and have been expanded through efforts to develop novel base pairs,^271^ or through the development XNA synthesis methods beyond polymerase-catalysed synthesis.^272^

Beyond the research itself, we must standardize publication criteria, by clearly describing selection protocols that are rigorous and reproducible, transparently detailing our NGS data analysis pipelines, and report our candidate selection methodologies.^273^

Solutions to these problems are possible and given the fast pace of research in the field, likely to emerge still within this decade. We look forward to see how the field will evolve and whether aptamers can finally live up to their potential.

## Conflicts of interest

There are no conflicts to declare.

## Data Availability

Our review makes reference to many published reports, but we have not included any original data or analyses in the current manuscript. As such, there are no data that can be made available.
